# Does the Timing of Antagonist Treatment Influence Cycle Outcomes in Unexpected Low Responders of POSEIDON Class 1 and 2?

**DOI:** 10.3390/jcm14061901

**Published:** 2025-03-12

**Authors:** Nina Medić, Damir Roje, Marina Šprem Goldštajn

**Affiliations:** 1 Department of Obstetrics and Gynecology, Clinical Hospital Sveti Duh, 10000 Zagreb, Croatia; 2 Department of Obstetrics and Gynecology, Split University Hospital Center, 21000 Split, Croatia; damir.roje@hormona.hr; 3 Department of Obstetrics and Gynecology, School of Medicine, University of Split, 21000 Split, Croatia; 4 Department of Obstetrics and Gynecology, Clinical Hospital Center, 10000 Zagreb, Croatia; marina.goldstajn@gmail.com

**Keywords:** POSEIDON classification, poor responder, IVF, ICSI, POSEIDON group 1, POSEIDON group 2, ART, GnRH antagonist

## Abstract

**Background/Objectives**: Unexpected low responders are patients with normal ovarian reserve tests who exhibit suboptimal responses to stimulation but have promising treatment potential due to adequate follicle availability. This study aimed to compare the live birth rates (LBRs) between fixed and flexible gonadotropin-releasing hormone antagonist (GnRH-ant) protocols in low-prognosis patients from Patient-Oriented Strategies Encompassing IndividualizeD Oocyte Number (POSEIDON) groups 1 and 2. **Methods**: This retrospective cohort study included 117 women classified as POSEIDON groups 1 and 2 who underwent GnRH-ant protocols for in vitro fertilization (IVF)/Intracytoplasmic Sperm Injection (ICSI) at the Petrova Maternity Hospital in Zagreb (2019–2020). The primary outcome analyzed was the live birth rate (LBR). The secondary outcomes were the GnRH start day, duration of gonadotropin therapy, number of oocytes, number of embryos, number of blastocysts, number of third-day embryos, number of vitrified embryos, positive pregnancy test, clinical pregnancy, and miscarriage rate. Group comparisons were conducted using Mann–Whitney and chi-squared tests. **Results**: In POSEIDON group 1, the fixed protocol significantly improved outcomes, with higher rates of clinical pregnancy, 12-week ongoing pregnancy, and LBRs (58.8% vs. 8.3%). More fresh transferable embryos were also noted (*p* < 0.05). In POSEIDON group 2, no significant differences were observed between protocols for any outcomes. **Conclusions**: The fixed GnRH-ant protocol improved pregnancy outcomes for POSEIDON group 1 but showed no advantage over the flexible protocol in POSEIDON group 2.

## 1. Introduction

The primary goal of modern assisted reproductive technology (ART) is to achieve the live birth of a healthy singleton baby in the shortest possible time, using effective and safe treatment strategies [1]. While ART success rates have significantly improved over the years, managing patients classified as poor ovarian responders (PORs) remains a complex and debated challenge, as no universally effective treatment strategies have been established [2].

In the past decade, efforts have been made to standardize the diagnosis of PORs [3]. The Bologna Criteria represented the first attempt to define POR systematically [4]. According to these criteria, for a woman to be classified as a POR patient, at least two of the following three criteria had to be present to establish the diagnosis: (1) advanced maternal age (≥40 years) or any other POR risk factor; (2) previous poor ovarian response (≤3 oocytes retrieved with a conventional protocol or previous cycle cancelled); and (3) abnormal ovarian reserve tests (antral follicle count [AFC] less than 5–7 follicles or anti-Müllerian hormone [AMH] below 0.5–1.1 ng/mL). Two episodes of PORs after maximal stimulation were deemed sufficient to classify a patient as a POR even in the absence of the other criteria mentioned. However, these criteria have faced criticism for grouping patients with diverse biological profiles and reproductive potential, leading to heterogeneous populations and limited practical guidance for managing these patients [3,4,5,6,7]. To address the limitations and heterogeneity within POR classifications under the Bologna Criteria, the Patient-Oriented Strategies Encompassing IndividualizeD Oocyte Number (POSEIDON) criteria was introduced [8].

This classification divides patients into four distinct low-prognosis groups based on ovarian reserve markers such as AMH, AFC, female age, and, if available, the number of oocytes retrieved in a previous conventional ovarian stimulation (COS) cycle. By integrating these parameters, the POSEIDON classification differentiates between two primary categories: expected poor ovarian responders (groups 3 and 4) and unexpected poor ovarian responders (groups 1 and 2).

The POSEIDON classification distinguishes between patients with adequate versus poor ovarian reserve and further stratifies them based on age and ovarian response. Group 1 includes patients younger than 35 years with adequate ovarian reserve (AFC ≥ 5 or AMH ≥ 1.2 ng/mL) who demonstrate an unexpected poor ovarian response (<4 oocytes retrieved, subgroup 1a) or a suboptimal response (4–9 oocytes retrieved, subgroup 1b). Group 2 consists of patients aged 35 years or older with adequate ovarian reserve (AFC ≥ 5 or AMH ≥ 1.2 ng/mL) who also experience an unexpected poor (<4 oocytes retrieved, subgroup 2a) or suboptimal (4–9 oocytes retrieved, subgroup 2b) ovarian response. POSEIDON groups 1 and 2 patients (the unexpected poor ovarian responders) are those with adequate ovarian reserve parameters before treatment but who had a low response to ovarian stimulation in terms of fewer follicles developed and fewer oocytes retrieved than expected from the ovarian reserve biomarkers. Normal ovarian reserve biomarkers according to the POSEIDON criteria are AMH level of ≥1.2 ng/mL and AFC ≥ 5. This results in a lower cumulative live birth rate (CLBR) per initiated cycle compared to normal responders [9,10,11]. The primary distinction between these two groups is age, which correlates with the expected rate of oocyte and embryo aneuploidy [12]. POSEIDON group 3 includes patients younger than 35 years with poor ovarian reserve parameters (AFC < 5 or AMH < 1.2 ng/mL), whereas group 4 consists of patients aged 35 years or older with poor ovarian reserve (AFC < 5 or AMH < 1.2 ng/mL). This classification provides a standardized framework for identifying and characterizing low-prognosis patients in assisted reproductive technologies and facilitates tailored treatment strategies to improve outcomes.

The pathophysiology of unexpected poor ovarian response remains unclear but appears to be primarily associated with polymorphisms in follicle-stimulating hormone (FSH) and luteinizing hormone (LH) receptors or variations in circulating endogenous LH levels [13,14,15]. In addition to genetic factors, suboptimal gonadotropin dosing, asynchronous follicular development during ovarian stimulation (OS), and technical issues related to ovulation triggering or oocyte retrieval are also proposed as contributing factors [16,17].

Ovarian stimulation is a critical component of ART, with outcomes largely dependent on the ovary’s sensitivity to gonadotropin stimulation. The accurate prediction of ovarian response is essential for optimal and individualized patient management [16]. While AFC and AMH are considered the most reliable predictors of ovarian potential, they cannot fully account for variations in individual responses to stimulation [17].

As a result, managing patients in POSEIDON groups 1 and 2 requires tailored diagnostic and therapeutic strategies distinct from those used for patients with impaired ovarian reserve [1]. These individuals may benefit significantly from pharmacological interventions, including a personalized approach to ovarian stimulation strategies. This involves selecting the appropriate type and dose of gonadotropins, optimizing the mode of pituitary suppression, and tailoring ovulation trigger protocols, aligning with the principles of individualized medicine [18,19,20].

Pituitary suppression is a crucial component of OS strategies. Since their introduction, gonadotropin-releasing hormone (GnRH) antagonists have gained widespread popularity among clinicians and are considered an equivalent alternative to long and short GnRH agonist protocols for poor ovarian response patients [21]. These antagonists offer several advantages, including convenience, flexibility, fewer side effects, reduced gonadotropin consumption, and shorter treatment duration [22,23].

The multidose GnRH antagonist protocol can be implemented in two ways: the fixed or flexible regimen. In the fixed regimen, GnRH antagonists are initiated on day 5 or 6 of OS, whereas in the flexible regimen, they start when the leading follicle reaches a diameter of 12–14 mm. However, data on which regimen is superior remain conflicting [24,25,26]. A recent systematic review by Venetis et al. comparing various GnRH antagonist initiation protocols found the fixed regimen to be significantly superior to the flexible protocol in terms of ongoing pregnancy rates [27].

The POSEIDON concept emphasizes an individualized approach to managing low-prognosis patients to optimize in vitro fertilization (IVF) success [28]. The antagonist protocol is the preferred pituitary suppression strategy for patients classified as POSEIDON groups 1 and 2 [7,28]. Despite this, there is limited evidence on whether the fixed or flexible mode of antagonist initiation offers better outcomes in terms of pregnancy and live birth rates for these patients.

In light of these considerations, we conducted this study to determine whether the mode of antagonist initiation, fixed or flexible, affects cycle outcomes, particularly ongoing pregnancy rates and live birth rates, in patients classified as POSEIDON group 1 and group 2.

## 2. Materials and Methods

### 2.1. Study Design

This was a single-centre, retrospective, cohort, observational study of women who underwent ART at Maternity Hospital Petrova, Zagreb, Croatia, between January 2019 and February 2020. We have analyzed 419 cycles where a multidose antagonist protocol was used. Cycles with inconsistent data and those performed for fertility preservation were excluded. Ethical approval was provided by the Ethical Committee of the Clinical Hospital Center Zagreb, Croatia (Class 8.1-24/196-2, No. 02/013 AG).

### 2.2. Study Population

Among 322 patients, only those who met the POSEIDON criteria were further evaluated. Patients were divided into four groups according to the POSEIDON classification, and only those classified as POSEIDON groups 1 and 2 (n = 117) were included in the current study. Patients belonging to POSEIDON groups 1 and 2 were then subdivided according to the preferred antagonist regimen, fixed or flexible, and only those who had undergone embryo transfer were compared.

The primary outcome was the live birth rate (LBR). The secondary outcomes were the number of oocytes, number of MII oocytes, number of embryos, number of blastocysts, 3rd-day embryos, number of vitrified embryos, positive pregnancy test, clinical pregnancy, miscarriage rate, GnRH start day, duration of antagonist therapy, and duration of gonadotropin therapy.

Clinical and treatment data were obtained from the hospital’s electronic medical records.

Ovarian stimulation was performed using recombinant follicle-stimulating hormone (recFSH) (Gonal-f, Merck Serono S.p.A., Bari, Italy), highly purified human menopausal gonadotropin (hMG) (Menopure, Ferring GmbH, Kiel, Germany), recFSH combined with hMG, or recFSH combined with recombinant luteinizing hormone (recLH) (Pergoveris, Merck Serono S.p.A., Bari, Italy) in a 2:1 ratio (recFSH: recLH).

Gonadotropin doses ranged from 150 to 300 IU and were adjusted based on the ovarian response. A daily dose of Cetrorelix 0.25 mg (Cetrotide, Merck Europe B.V., Amsterdam, The Netherlands) was administered on the 6th day of stimulation for women treated with the fixed protocol and when the dominant follicle reached a mean diameter of 13–14 mm for women treated with the flexible protocol.

Final oocyte maturation was induced using human chorionic gonadotropin (hCG) (Ovitrelle 250 µg, Merck Serono S.p.A., Amsterdam, The Netherlands), and oocyte retrieval was performed 36 h later. Oocytes were inseminated via IVF or Intracytoplasmic Sperm Injection (ICSI). Zygotes were cultured until day 3 or the blastocyst stage. Embryo transfer (ET) was performed under ultrasound guidance, and surplus embryos were vitrified. Luteal phase support was provided with daily intravaginal progesterone administration. A pregnancy test was performed two weeks after the ET.

### 2.3. Statistical Analysis

Statistical analysis was performed using SPSS software, version 22.0. Continuous variables are represented as medians with a first quartile (Q1) and third quartile (Q3) and were compared using the Mann–Whitney U test to assess the differences between the groups. Categorical variables are represented as the absolute frequency (N) and relative frequency (%) and were compared using the chi-squared test. Additionally, adjusted odds ratios with 95% confidence intervals (CIs) were calculated. The level of statistical significance was set at 0.05.

## 3. Results

**Figure 1:**
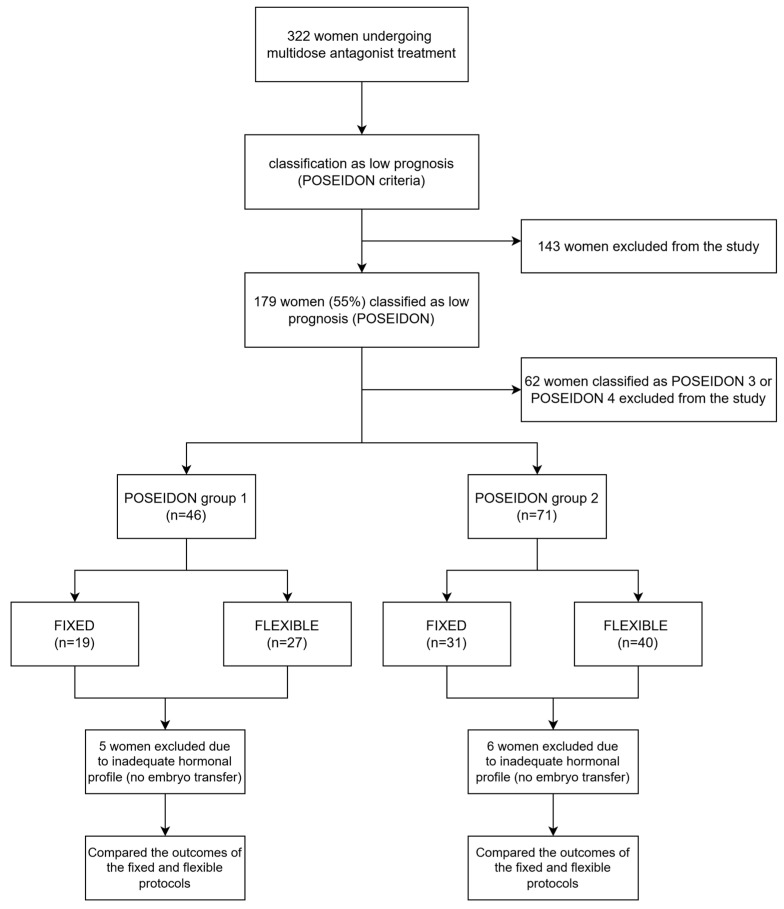
Flowchart of the study population. A total of 322 women undergoing multi-dose antagonist treatment were evaluated, with 179 women (55%) classified as low prognosis according to the POSEIDON criteria. These women were divided into POSEIDON group 1 (n = 46) and POSEIDON group 2 (n = 71) based on their specific characteristics. Each group was further divided into fixed and flexible GnRH antagonist protocols. Women who did not undergo embryo transfer due to an inadequate hormonal profile (5 in POSEIDON group 1 and 6 in POSEIDON group 2) were excluded from the analysis. The outcomes of the fixed and flexible protocols were then compared within each group.

Among 322 women who underwent multi-dose antagonist treatment, 179 of them (55%) were classified as having a low prognosis according to the POSEIDON criteria. A total of 117 (65%) women were classified as either POSEIDON group 1 (n = 46) or as POSEIDON group 2 (n = 71). Patients classified as POSEIDON group 1 were younger than 35 years, had adequate ovarian reserve parameters (AMH ≥ 1.2 ng/mL and AFC ≥ 5), and retrieved fewer than 10 oocytes in a stimulated cycle. Those who retrieved fewer than 4 oocytes were classified as subgroup 1a, while those who retrieved 4–9 oocytes were classified as subgroup 1b. Patients classified as POSEIDON group 2 were 35 years or older, had adequate ovarian reserve parameters (AMH ≥ 1.2 ng/mL and AFC ≥ 5), and retrieved fewer than 10 oocytes in a stimulated cycle. Those who retrieved fewer than 4 oocytes were classified as subgroup 2a, while those who retrieved 4–9 oocytes were classified as subgroup 2b. In POSEIDON group 1, 19 (41.3%) women were treated with fixed and 27 (58.7%) with the flexible antagonist regimen. In POSEIDON group 2, 31 (43.7%) women were treated with fixed and 40 (56.3%) with the flexible regimen. Five women classified as POSEIDON 1 and six women classified as POSEIDON 2 did not have embryo transfer due to the inadequate hormonal profile. Only those who had undergone embryo transfer were further evaluated in the study. An overview of the study design is shown in Figure 1.

We compared the baseline characteristics of POSEIDON groups 1 and 2, finding statistically significant differences only in age, the duration of infertility, the number of previous cycles, and the level of FSH. Other parameters, including levels of LH, estradiol (E2), AMH, the basal value of progesterone (P4), the value of P4 on hCG day, and AFC, showed no significant differences (Table 1).

### 3.1. POSEIDON Group 1

In POSEIDON group 1, no statistically significant differences were observed between the fixed and flexible protocols in terms of the total number of retrieved oocytes, number of metaphase II (MII) oocytes, gonadotropin consumption, duration of gonadotropin therapy, or endometrial thickness on the day of hCG administration (Table 2).

Furthermore, no statistically significant differences were observed between the fixed and flexible GnRH antagonist protocols in several parameters, including embryo transfers involving 1BC (17.6% vs. 41.7%), the number of KRIO embryos (23.5% vs. 8.3%) for both 1BC and 2BC, and the total number of transferred blastocysts, including fresh and frozen transfers (*p* > 0.05 for all comparisons, Table 3).

However, the analysis revealed significant differences between the fixed and flexible GnRH antagonist protocols in several parameters. The number of fresh transferred embryos was significantly higher in the flexible group (36 vs. 31, *p* < 0.05), while the total number of transferred embryos on day 3 was greater in the fixed group (16 vs. 6, *p* < 0.05). In POSEIDON group 1, a positive βhCG was observed in more than half of the cycles for women treated with the fixed regimen (64.7%) compared to 33.3% in those treated with the flexible regimen. Biochemical pregnancies were twice as common in the flexible regimen compared to the fixed regimen (12.5% vs. 5.8%). Women treated with the fixed regimen demonstrated significantly better pregnancy outcomes, including clinical pregnancy, 12-week ongoing pregnancy, and live birth rates. Clinical pregnancy rates were significantly higher in the fixed group compared to the flexible group (58.82% vs. 20.83%, *p* < 0.05). Additionally, the proportion of pregnancies continuing beyond 12 weeks was significantly greater in the fixed group (58.82% vs. 12.5%, *p* < 0.05). The pregnancy rate in the fixed regimen group was 64.7%, with a live birth rate (LBR) of 58.8% (95% CI: 35.4–82.2%). In contrast, the pregnancy rate in the flexible regimen group was 33.3%, with a live birth rate of 8.3% (95% CI: 0.0–19.4%). These findings underscore the impact of protocol type on embryo transfer and pregnancy outcomes and are presented in Table 3.

We also conducted a subgroup analysis of pregnant women in POSEIDON groups 1 and 2 to compare clinical and cycle outcome characteristics between those treated with the fixed and flexible GnRH antagonist protocols. A subgroup analysis of pregnant women in POSEIDON group 1 (Supplementary Table S1) revealed that those treated with the flexible protocol experienced miscarriage at twice the rate of those on the fixed regimen.

Additionally, we performed a separate subgroup analysis of POSEIDON groups 1 and 2 to compare clinical and pregnancy outcomes between pregnant and non-pregnant women treated with either the fixed or flexible GnRH antagonist protocol. In this broader subgroup analysis, including both pregnant and non-pregnant women in POSEIDON group 1 (Supplementary Table S2), we found that the number of obtained blastocysts was significantly higher in the pregnant group, regardless of the protocol used.

### 3.2. POSEIDON Group 2

Patients classified as POSEIDON group 2 underwent the same evaluation, comparing identical cycle characteristics between the fixed and flexible protocols (data presented in Table 4 and Table 5). Both regimens yielded similar results for most parameters, including the duration of infertility, number of IVF procedures, gonadotropin dose, number of preovulatory follicles, endometrial thickness on the day of hCG, hormone levels (P4 and E2) on the day of hCG, and the number of oocytes and MII oocytes retrieved (*p* > 0.05 for all, Table 4). While differences were observed in the duration of stimulation, timing of GnRH-antagonist initiation, and duration of GnRH-antagonist use, these parameters were not primary outcomes of interest and therefore do not impact the overall comparability of the two regimens in this group.

Furthermore, in POSEIDON group 2, both the fixed and flexible GnRH antagonist protocols yielded similar results across most parameters, including the number of fresh transferred embryos, the total number of blastocysts transferred (fresh and frozen), the number of embryos transferred on day 3, βhCG positivity rates, and clinical pregnancy rates (*p* > 0.05 for all, Table 5). However, women treated with the flexible regimen had significantly more single blastocyst transfers compared to those in the fixed regimen (36.1% vs. 3.4%, *p* < 0.01). Despite this difference, key pregnancy outcomes, including live birth rates, did not significantly differ between the two protocols.

Furthermore, we extended our analysis to POSEIDON group 2 (Supplementary Table S3), comparing clinical and pregnancy outcomes between pregnant and non-pregnant women treated with the fixed and flexible protocols, as we did for POSEIDON group 1. No significant differences were observed in cycle characteristics between pregnant and non-pregnant women in either protocol group.

## 4. Discussion

To the best of our knowledge, this is the first study to explore the impact of antagonist regimen choice on the live birth rate in patients classified as POSEIDON groups 1 and 2.

Our study demonstrated that for patients classed as POSEIDON 1, the administration of antagonist treatment in a standard fixed scheme significantly improved the live birth rate compared to individualized flexible administration. The reason could be attributed to its impact on oocyte–embryo quality and/or endometrial receptivity [29,30].

The delivery rate is closely associated with the number of oocytes retrieved during OS [3]. Although a similar number of mature oocytes were obtained in both groups, patients treated with the fixed protocol in POSEIDON group 1 obtained a higher number of embryos with a statistically higher number of embryos available for transfer in a fresh cycle. More embryos represent a better selection and therefore may increase the chance of pregnancy [31]. According to Chen et al., the number of transferable embryos is one of the independent predictive factors for live birth in unexpected poor ovarian response patients [32].

Blastocyst embryo transfer is a favourable outcome of controlled ovarian stimulation for all POSEIDON groups, as blastocysts are believed to have the highest implantation potential [33,34]. The implantation potential of euploid blastocysts remains consistent across all age groups and results in a healthy live birth in approximately 50% of cases, regardless of the woman’s age [33]. In our study, women treated with the flexible antagonist protocol underwent blastocyst transfers in over 80% of cases, whereas those treated with the fixed protocol had a more balanced distribution of blastocyst and day-3 embryo transfers. Despite this, women treated with the fixed antagonist protocol achieved pregnancy twice as often, with live births occurring in more than half of these cases. In contrast, the flexible protocol yielded a live birth rate (LBR) of less than 10%.

In the fixed protocol group, we sought to identify factors associated with pregnancy. Our analysis revealed that the number of obtained blastocysts was significantly higher in the pregnancy group.

It has been hypothesized that the endocrine environment during the early follicular phase in antagonist cycles may influence reproductive outcomes [30]. Kolibianakis et al. suggested that the exposure of the genital tract to both the luteinizing hormone and estradiol (E2) before the initiation of antagonist treatment could differentiate between pregnant and nonpregnant patients [29].

Based on these findings, we speculate that the fixed regimen may create a more favourable endocrine environment and that transferring embryos at the blastocyst developmental stage could be associated with the highest likelihood of achieving pregnancy.

In a subgroup analysis of all pregnant women in POSEIDON group 1, we compared stimulation and cycle outcome characteristics between the fixed and flexible protocols. Both groups demonstrated similar stimulation characteristics, with the exception that women treated with the flexible protocol had significantly higher FSH consumption compared to those in the fixed protocol. Despite these similarities, their clinical outcomes differed significantly. Nearly 80% of women treated with the individualized flexible protocol experienced miscarriage, whereas women treated with the fixed protocol achieved live births twice as often—a statistically significant difference.

We may speculate that initiating antagonist treatment after five days of gonadotropin stimulation could enhance follicular synchronization, thereby creating a more favourable endocrine environment for oocyte maturation and development. Delaying antagonist administration allows LH levels to remain unsuppressed, leading to an earlier rise in estradiol (E2) levels [29]. However, elevated E2 levels could have potentially detrimental effects on pregnancy rates, primarily by reducing endometrial receptivity in fresh cycles or causing the over-maturation of oocytes [35,36,37,38].

An earlier increase in estradiol (E2) levels induces the premature appearance of progesterone-binding receptors, facilitating the earlier initiation of endometrial transformation. In controlled ovarian stimulation (cOS), this earlier rise in estradiol is often linked to higher recombinant FSH (recFSH) consumption or the delayed administration of GnRH antagonists [39]. The endometrial advancement of more than three days relative to the expected chronological date at the time of oocyte retrieval is significantly associated with a reduced likelihood of pregnancy [40]. Recent data suggest that endometrial advancement at oocyte retrieval is almost universally observed in ART cycles using recFSH, GnRH antagonists, or hCG as the trigger [41].

Although no significant difference in implantation rates was observed between the fixed and flexible groups, the miscarriage rate was twice as high in the flexible group, potentially linked to a transformed endocrine environment of the endometrium.

In POSEIDON group 2, no differences were observed between the two protocols regarding the number of transferable embryos, pregnancy rates, or live birth rates. The two protocols differed significantly in the timing of antagonist initiation, duration of antagonist therapy, and gonadotropin treatment. Patients treated with the fixed protocol had a longer duration of gonadotropin and GnRH antagonist treatment, as well as an earlier start of antagonist therapy. These findings align with the expected characteristics of this type of pituitary suppression protocol [22].

The results of the current study align with recently published data on the preferable antagonist regimen for women of advanced maternal age. According to that study, there were no significant differences in the pregnancy outcomes between women undergoing fixed or flexible GnRH antagonist protocols, although the fixed protocol demonstrated a trend of superiority over the flexible protocol [42].

In our study, comparable variables related to pregnancy outcomes were similar between the two groups. However, when analyzing factors associated with pregnancy within each group, we found that the number of transferable embryos was significantly higher in women who became pregnant and were treated with the flexible antagonist regimen.

In a study by Depalo et al., conducted among normo-ovulatory women younger than 39 with a mean age of 35, pregnancy outcomes were not influenced by the type of GnRH antagonist protocol used. The study highlighted that a lower trend in LH concentration from baseline to the end of stimulation, younger age, and a larger pool of recruitable follicles were key factors associated with pregnancy success [38,43].

GnRH antagonists are approved for use in assisted reproduction to prevent premature LH surges [23]. They can be initiated on either day 6 of stimulation (fixed regimen) or when the leading follicle reaches a diameter of 12–14 mm (flexible regimen). Both protocols are effective in preventing LH surges and achieving approximately a 30% pregnancy rate per embryo transfer. However, the question of which protocol offers better pregnancy outcomes remains a topic of debate, with no clear consensus established [27,44].

Controlled ovarian stimulation is a cornerstone of assisted reproductive technology. The response to gonadotropin stimulation varies widely and is influenced by individual patient characteristics [17]. Multifollicular growth is essential in modern IVF to obtain an adequate number of oocytes. However, during stimulation, not all follicles develop synchronously, and their hormonal profiles differ. Ovarian sensitivity to stimulation depends on the number of follicles responsive to FSH [16].

In GnRH antagonist protocols, the absence of initial pituitary suppression allows for a more diverse hormonal milieu during early follicular recruitment [29,30].

We hypothesize that greater hormonal fluctuations may occur in younger women due to their naturally higher number of follicles responsive to FSH stimulation. Consequently, varying endocrine profiles during the early follicular phase may influence the overall outcome of the stimulation cycle.

We speculate that administering GnRH antagonists using the standard fixed regimen may facilitate better follicle synchronization, thereby creating a more uniform endocrine environment for the development of the follicular cohort and adequate endometrial preparation.

The POSEIDON classification aims to provide individualized treatment tailored to the specific characteristics of each patient, maximizing IVF success. Based on the results of our study, we propose that the fixed antagonist protocol may be the preferred choice for patients classified as POSEIDON group 1. For POSEIDON group 2 patients, the choice of GnRH antagonist strategy could be made more flexibly, depending on the clinical context and patient preferences.

The limitations of this study include its retrospective observational design, which carries an inherent risk of unknown confounders affecting outcomes. Additionally, the small sample size within groups limits the generalizability of our findings. Another potential source of bias in the flexible regimen is the initiation of antagonists based on ultrasound measurements of the leading follicle, which may be subject to interobserver variability.

## 5. Conclusions

The current study identified a statistically significant difference in the live birth rate (LBR) per embryo transfer (ET) between women treated with fixed and flexible regimens in POSEIDON group 1. Women who underwent the fixed antagonist protocol demonstrated better pregnancy outcomes, including higher clinical pregnancy and live birth rates, compared to those treated with the flexible protocol. In POSEIDON group 2, no significant differences were observed between the two protocols. As the POSEIDON classification aims to provide individualized management to maximize IVF success, further investigation through a randomized controlled trial with a larger sample size would be valuable to compare these two protocols.

## Figures and Tables

**Table 1:** Baseline and cycle characteristics of women classified as POSEIDON group 1 and POSEIDON group 2.

		POSEIDON Group 1			POSEIDON Group 2			*p*-Value
		Median	Q1	Q3	Median	Q1	Q3	
Age	33.00		30.00	34.00	38.00	37.00	40.00	**<0.05**
Duration of infertility	5.00		3.25	6.00	6.00	5.00	8.00	**<0.05**
No. of IVF	0.00		0.00	1.00	1.00	0.00	2.00	**<0.05**
FSH (IU/L)	6.30		5.40	7.38	7.20	5.85	8.55	**<0.05**
LH (IU/L)	4.00		3.03	5.88	5.10	3.80	6.10	>0.05
Estradiol (pmol/L)	183.00		136.50	210.25	129.00	104.00	179.50	>0.05
Basal value of P4 (nmol/L)	0.90		0.90	1.35	1.00	0.88	1.30	>0.05
P4 on hCG day (nmol/L)	1.75		1.425	2.35	2.25	1.40	2.70	>0.05
AMH (pmol/L)	13.00		9.50	20.40	12.35	9.43	15.75	>0.05
AFC	10.00		8.00	10.00	10.00	6.50	12.00	>0.05

Variables are represented as medians with first (Q1) and third quartiles (Q3) and were compared using the Mann–Whitney U test. Statistically significant differences are indicated in bold. AFC, antral follicle count; AMH, anti-Müllerian hormone; FSH, follicle-stimulating hormone; hCG, human chorionic gonadotropin; LH, luteinizing hormone; IVF, in vitro fertilization; P4, progesterone.

**Table 2:** Clinical outcomes of women treated with fixed and flexible GnRH antagonist protocols in patients classified as POSEIDON group 1.

Group	POSEIDON Group 1						*p*-Value
Cycle Charachteristics	Fixed (n = 19)			Flexible (n = 27)			
Parameters	Median	Q1	Q3	Median	Q1	Q3	
Duration of infertility	5	3.5	6	5	4	6	>0.05
No. of IVF procedures	0	0	1	0	0	1	>0.05
Gonadotropin consumption (IU)	2400	1800	2962.5	2812	2043.75	3000	>0.05
Duration of stimulation	10	9	12	10	9	12	>0.05
Start of GnRH-ant Day	7	7	7	8	7	9	**<0.05**
Duration of GnRH-ant therapy	5	4	6	4	4	5	>0.05
Number of preovulatory follicles	7	5	8	6	5	8.25	>0.05
Endometrial thickness on the Day of hCG (mm)	9.5	9	11.175	10.1	9.5	11	>0.05
Value of P4 on the day of HCG (nmol/L)	2.65	1.725	3.425	1.6	0.95	2.05	>0.05
E2 on the day of hCG (pmol/L)	3080	2711.5	4129	2528.5	1971.5	3076.5	>0.05
E2 on the day of hCG (pg/mol)	839	738	1124	637	529	831	>0.05
No. of oocytes	5	4	7	6	4	7.5	>0.05
No. of MII oocytes	4	3	5.5	5	3	6	>0.05

Variables are described as medians with first (Q1) and third quartiles (Q3) and were compared using the Mann–Whitney U test. Statistically significant differences are indicated in bold. E2, estradiol; GnRH-ant, gonadotropin-releasing hormone antagonist; hCG, human chorionic gonadotropin; IVF, in vitro fertilization; MII, metaphase II; P4, progesterone.

**Table 3:** Laboratory and pregnancy outcomes of women treated with fixed and flexible GnRH antagonist protocols in patients classified as POSEIDON group 1.

Group	POSEIDON Group 1				
Cycle	Fixed (n = 17)		Flexible (n = 24)		
Parameters	f	p (%)	f	p (%)	*p*-Value
ET-BC, 1BC or 2BC (yes/no)	9	52.9%	20	83.3%	>0.05
ET 1BC	3	17.6%	10	41.7%	>0.05
KRIO (n,%)	4	23.5%	2	8.3%	>0.05
ET 2BC	6	35.3%	10	41.7%	>0.05
KRIO (n,%)	4	23.5%	2	8.3%	>0.05
No. of fresh transferred embryos	31		36		**<0.05**
No. of fresh transferred BC	15		30		>0.05
No. of all BC (ET + FET)	20		33		>0.05
No. of ET embryos 3rd day	8	47.1%	4	16.7%	>0.05
ET of 1 embryo 3rd day	0	0.0%	2	8.3%	>0.05
ET of 2 embryos 3rd day	8	47.1%	2	8.3%	**<0.05**
Total No. of transferred embryos 3rd day	16		6		**<0.05**
βhCG+	11	64.7%	8	33.3%	>0.05
Clinical pregnancy	10	58.82%	5	20.83%	**<0.05**
12 weeks	10	58.82%	3	12.5%	**<0.05**
+12 weeks abortion	0	0.0%	2	8.3%	>0.05
Missed abortion	0	0.0%	3	12.5%	>0.05
Biochemical pregnancy	1	5.8%	3	12.5%	>0.05
Live birth	10	58.8%	2	8.3%	**<0.01**

Categorical and count variables are represented as absolute frequency (f) and relative frequency (p, %) and were compared using the chi-square test. Statistically significant differences are indicated in bold. BC, blastocyst; BhCG, positive beta-human chorionic gonadotropin (βHCG) test; ET, embryo transfer; FET, frozen embryo transfer.

**Table 4:** Clinical outcomes of women treated with fixed and flexible GnRH antagonist protocols in patients classified as POSEIDON group 2.

Group	POSEIDON Group 2						*p*-Value
Cycle Charachteristics	Fixed (n = 31)			Flexible (n = 40)			
Parameters	Median	Q1	Q3	Median	Q1	Q3	
Duration of infertility	6	5	6	7	5	9	>0.05
No. of IVF procedures	1	0	2	1	0	2	>0.05
Gonadotropin consumption (IU)	2250	2025	2925	2400	2025	2550	>0.05
Duration of stimulation	11	10	11	10	8.75	11	**<0.01**
Start of GnRH-ant Day	7	7	7	8	7	9	**<0.05**
Duration of GnRH-ant therapy	5	4	6	4	3	4	**<0.05**
Number of preovulatory follicles	7	6	7	5	5	7	>0.05
Endometrial thickness on the Day of hCG (mm)	11.5	9.7	13	9.8	8.75	11.25	>0.05
Value of P4 on the day of HCG (nmol/L)	2.3	1.6	2.6	1.8	1.3	2.7	>0.05
E2 on the day of hCG (pmol/L)	5026	29,997.75	6298.5	4857.5	3022.5	6430.5	>0.05
E2 on the day of hCG (pg/mol)	1509	957	1644.25	1376.5	855.75	1878.95	>0.05
No. of oocytes	6	4	7	5	4	7	>0.05
No. of MII oocytes	4	3	6	4	3	6	>0.05

Variables are described as medians with first (Q1) and third quartiles (Q3) and were compared using the Mann–Whitney U test. Statistically significant differences are indicated in bold. E2, estradiol; GnRH-ant, gonadotropin-releasing hormone antagonist; hCG, human chorionic gonadotropin; IVF, in vitro fertilization; MII, metaphase II; P4, progesterone.

**Table 5:** Laboratory and pregnancy outcomes of women treated with fixed and flexible GnRH antagonist protocols in patients classified as POSEIDON group 2.

Group	POSEIDON Group 2				
Cycle	Fixed (n = 31)		Flexible (n = 40)		*p*-Value
Parameters	f	p (%)	f	p (%)	
ET-BC, 1BC or 2BC (yes/no)	12	41.4%	21	58.3%	>0.05
ET 1BC	1	3.4%	13	36.1%	**<0.01**
KRIO (n,%)	4	13.8%	4	11.1%	>0.05
ET 2BC	10	34.5%	8	22.2%	>0.05
KRIO (n,%)	4	13.8%	4	11.1%	>0.05
No. of fresh transferred embryos	48		52		>0.05
No. of fresh transferred BC	23		29		>0.05
No. of all BC (ET + FET)	30		39		>0.05
No. of ET embryos 3rd day	17	58.6%	13	36.1%	>0.05
ET of 1 embryo 3rd day	6	20.7%	3	8.3%	>0.05
ET of 2 embryos 3rd day	11	37.9%	10	27.8%	>0.05
Total No. of transferred embryos 3rd day	28		23		>0.05
βhCG+	8	27.5%	9	25%	>0.05
Clinical pregnancy	7	24.1%	9	25%	>0.05
12 weeks	4	13.7%	5	13.8%	>0.05
+12 weeks abortion	0	0.0%	0	0.0%	>0.05
Missed abortion	4	13.7%	4	11.11%	>0.05
Biochemical pregnancy	0	0.0%	0	0.0%	>0.05
Live birth	4	13.79%	5	13.88%	>0.05

Categorical and count variables are represented as absolute frequency (f) and relative frequency (p, %) and were compared using the chi-square test. Statistically significant differences are indicated in bold. BC, blastocyst; 1 BC, one blastocyst, 2 BC, two blastocysts, BhCG, positive beta-human chorionic gonadotropin (βHCG) test; ET, embryo transfer; KRIO, frozen embryo; FET, frozen embryo transfer.

## Data Availability

The data presented in this study are available on request from the corresponding author. Neither Research Ethics Committee approval nor consent from individual participants have been given to permit the open release of the individual-level research data underlying this study. The datasets analyzed during the current study are therefore not publicly available.

## Supplementary Materials

The following supporting information can be downloaded at: https://www.mdpi.com/article/10.3390/jcm14061901/s1.
