# Supplementary material for: Does the Timing of Antagonist Treatment Influence Cycle Outcomes in Unexpected Low Responders of POSEIDON Class 1 and 2?
Source: J Clin Med. 2025 Mar 12;14(6):1901. doi: 10.3390/jcm14061901 (PMC11942690; doi:10.3390/jcm14061901)
Supplement: Supplementary file 1 [file jcm-14-01901-s001.zip › jcm-3395791-supplementary.pdf]

Supplementary File

Supplementary Table S1. Clinical and pregnancy outcomes among pregnant women classified as POSEIDON group 1 and 2 who were treated with the fixed and flexible GnRH antagonist protocol.

**Group**	**POSEIDON group 1**					**POSEIDON group 2**				
**Protocol**	**Fixed (n = 19)**		**Flexible (n = 27)**			**Fixed (n = 31)**		**Flexible (n = 40)**		
**Cycle characteristics**	**Pregnant (n = 11)**		**Pregnant (n = 8)**		**p-value**	**Pregnant (n = 7)**		**Pregnant (n = 8)**		**p-value**
Duration of infertility (years)	2	[2, 3]	2	[2, 5]	>0.05	3	[2, 5]	4	[2, 5]	>0.05
Gonadotropin consumption (IU)	1950	[1800, 2700]	2700	[2100, 3000]	**<0.05**	2400	[2025, 2700]	2437.5	[2325, 2475]	>0.05
Duration of stimulation (days)	4	36%	4	50%	>0.05	4	57%	6	75%	>0.05
Start of GnRH-ant Day	7	[6.5, 7]	7.5	[7, 9]	>0.05	7	[7, 7]	8	[8, 9]	**<0.05**
Duration of GnRH-ant therapy (days)	5	[4, 6]	4.5	[3.5, 5]	>0.05	5	[4, 6]	4	[3, 4.5]	>0.05
Number of preovulatory follicles	7	[5, 8]	5	[5, 8]	>0.05	7	[6.5, 7.5]	5	[5, 12]	>0.05
Endometrial thickness on the Day of hCG (mm)	10.1	[9, 12.4]	9.5	[9.5, 10]	>0.05	9.9	[9.3, 11.5]	12	[10, 12]	>0.05
No. of oocytes	7	[4, 7]	6	[3, 9]	>0.05	5	[4, 8]	5	[3.5, 7.5]	>0.05

No. of MII oocytes	5	[3, 7]	5	[2.5, 6]	>0.05	4.5	[4, 5]	5	[2, 7]	>0.05
4 oocytes (N, %)	5	45%	3	38%	>0.05	2	29%	4	50%	>0.05
4/9 oocytes (N, %)	6	55%	5	63%	>0.05	5	71%	4	50%	>0.05
ET 1 BC (N, %)	2	18%	3	38%	>0.05	0	0%	2	25%	>0.05
ET 2 BC (N, %)	5	45%	4	50%	>0.05	3	43%	3	38%	>0.05
ET -BC, 1BC or 2 BC (N, %)	7	64%	7	88%	>0.05	3	43%	5	63%	>0.05
ET 1 embryo 3rd day (N, %)	0	0%	0	0%	>0.05	0	0%	0	0%	>0.05
ET 2 embryos 3rd day (N, %)	4	36%	1	13%	>0.05	3	43%	3	38%	>0.05
KRIO (N, %)	3	27%	1	13%	>0.05	2	29%	2	25%	>0.05
KRIO 1BC or 2BC (N)	0	[0, 1]	0	[0, 0]	>0.05	0	[0, 2]	0	[0, 1]	>0.05
No. of fresh transferred embryos	2	[2, 2]	2	[1, 2]	>0.05	2	[2, 2]	2	[1.5, 2]	>0.05
No. of fresh transferred BC	1	[0, 2]	1.5	[1, 2]	>0.05	0	[0, 2]	1	[0, 2]	>0.05

No. of all BC (ET+FET)	1	[0, 2]	2	[1, 2]	>0.05	0	[0, 4]	1.5	[0, 2.5]	>0.05
Total no. of transferred embryos 3rd day	0	[0, 2]	0	[0, 0]	>0.05	0	[0, 2]	0	[0, 2]	>0.05
No. of ET embryos 3rd day	4	36%	1	13%	>0.05	3	43%	3	38%	>0.05
12 weeks pregnant (N, %)	10	91%	3	38%	**<0.05**	4	57%	5	63%	>0.05
+12 weeks missed abortion (N, %)	0	0%	2	25%	>0.05	4	57%	5	63%	>0.05
Missed abortion (N, %)	0	0%	3	38%	>0.05	3	43%	3	38%	>0.05
Biochemical pregnancy (N, %)	1	9%	3	38%	>0.05	3	43%	3	38%	>0.05
Live birth (N, %)	10	91%	2	25%	**<0.05**	4	57%	5	63%	>0.05
Clinical pregnancy rate	10	91%	5	63%	>0.05	6	86%	8	100%	>0.05

Categorical variables are represented as absolute frequency (N) and relative frequency (p, %) and were compared using the chi–square test. Continuous variables are described as medians with [first quartile (Q1), third quartile (Q3)], and were compared using the Mann–Whitney U test. Statistically significant differences are indicated in bold. BC, blastocyst; 1 BC, one blastocyst; 2BC, two blastocysts; ET, embryo transfer; KRIO, frozen embryo; FET, frozen embryo transfer; GnRH-ant, gonadotropin-releasing hormone antagonist; hCG, Human Chorionic Gonadotropin; MII, metaphase II.

Supplementary Table S2. Clinical and pregnancy outcomes among pregnant and non-pregnant women classified as POSEIDON group 1 who were treated with the fixed and flexible GnRH antagonist protocol.

**Group**	**POSEIDON group 1**									
**Protocol**	**Fixed (n = 19)**					**Flexible (n = 27)**				
**Cycle characteristics**	**Pregnant (n = 11)**		**Not pregnant (n = 8)**		**p-value**	**Pregnant (n = 8)**		**Not pregnant (n = 19)**		**p-value**
Duration of infertility (years)	2	[2, 3]	3	[2, 4]	>0.05	2	[2, 5]	2	[2, 3]	>0.05
Gonadotropin consumption (IU)	1,950.0	[1800, 2700]	2,850.0	[1912.5, 3337.5]	>0.05	2,700.0	[2100, 3000]	2,925.0	[1800, 3000]	>0.05
Duration of stimulation (days)	4	36%	6	75%	>0.05	4	50%	10	53%	>0.05
Start of GnRH-ant Day	7	[6.5, 7]	7	[7, 7]	>0.05	7.5	[7, 9]	8	[7, 9]	>0.05
Duration of GnRH-ant therapy	5	[4, 6]	5.5	[4, 6]	>0.05	4.5	[3.5, 5]	4	[4, 5]	>0.05
Number of pre-ovulatory follicles	7	[5, 8]	7	[5.5, 7.5]	>0.05	5	[5, 8]	7	[5, 9]	>0.05

Endometrial thickness on the Day of hCG (mm)	10.1	[9, 12.4]	9.4	[9, 9.9]	>0.05	9.5	[9.5, 10]	10.8	[9.6, 11]	>0.05
No. of oocytes	7	[4, 7]	4.5	[4, 6.5]	>0.05	6	[3, 9]	6	[4, 7]	>0.05
No. of MII oocytes	5	[3, 7]	3.5	[3, 4]	>0.05	5	[2.5, 6]	5	[3, 6]	>0.05
4 oocytes (N, %)	5	45%	4	50%	>0.05	3	38%	8	42%	>0.05
4/9 oocytes (N, %)	6	55%	4	50%	>0.05	5	63%	11	58%	>0.05
ET -BC, 1BC or 2 BC (N, %)	7	64%	2	25%	>0.05	7	88%	13	68%	>0.05
ET 1 BC (N, %)	2	18%	1	13%	>0.05	3	38%	7	37%	>0.05
ET 2BC (N, %)	5	45%	1	13%	>0.05	4	50%	6	32%	>0.05
ET 1 embryos 3rd day (N, %)	0	0%	0	0%	>0.05	0	0%	2	11%	>0.05
ET 2 embryos 3rd day (N, %)	4	36%	4	50%	>0.05	1	13%	1	5%	>0.05
KRIO (N, %)	3	27%	1	13%	>0.05	1	13%	1	5%	>0.05
KRIO 1BC or 2BC (N)	0	[0, 1]	0	[0, 0]	>0.05	0	[0, 0]	0	[0, 0]	>0.05
No. of fresh transferred embryos	2	[2, 2]	2	[0.5, 2]	>0.05	2	[1, 2]	1	[1, 2]	>0.05

No. of fresh transferred BC	1	[0, 2]	0	[0, 0.5]	>0.05	1.5	[1, 2]	1	[0, 2]	>0.05
No. of all BC (ET+FET)	1	[0, 2]	0	[0, 0.5]	**<0.05**	2	[1, 2]	1	[0, 2]	>0.05
Total No. of transferred embryos 3rd day	0	[0, 2]	1	[0, 2]	>0.05	0	[0, 0]	0	[0, 0]	>0.05
No. of ET embryos 3rd day	4	36%	4	50%	>0.05	1	13%	3	16%	>0.05

Categorical variables are represented as absolute frequency (N) and relative frequency (p, %) and were compared using the chi–square test. Continuous variables are described as medians with [first quartile (Q1), third quartile (Q3)], and were compared using the Mann–Whitney U test. Statistically significant differences are indicated in bold. BC, blastocyst; 1 BC, one blastocyst; 2BC, two blastocysts; ET, embryo transfer; KRIO, frozen embryo; FET, frozen embryo transfer; GnRH-ant, gonadotropin-releasing hormone antagonist; hCG, Human Chorionic Gonadotropin; MII, metaphase II.

Supplementary Table S3. Clinical and pregnancy outcomes among pregnant and non-pregnant women classified as POSEIDON group 2 who were treated with the fixed and flexible GnRH antagonist protocol.

**Group**	**POSEIDON group 2**									
**Protocol**	**Fixed (n = 31)**					**Flexible (n = 40)**				
	**Pregnant (n = 7)**		**Not pregnant (n = 24)**		**p-value**	**Pregnant (n = 8)**		**Not pregnant (n = 32)**		**p-value**
Duration of infertility (years)	3	[2, 5]	3	[2, 4]	>0.05	4	[2, 5]	4	[2, 5]	>0.05
Gonadotropin consumption (IU)	2400	[2025, 2700]	2250	[2025, 2925]	>0.05	2437.5	[2325, 2475]	2400	[2025, 2625]	>0.05
Duration of stimulation (days)	4	57%	10	42%	>0.05	6	75%	15	47%	>0.05
Start of GnRH-ant Day	7	[7, 7]	7	[7,7]	>0.05	8	[8, 9]	8	[7, 9]	>0.05
Duration of GnRH-ant therapy (days)	5	[4, 6]	5	[4,6]	>0.05	4	[3, 4.5]	4	[3, 4]	>0.05
Number of pre-ovulatory follicles	7	[6.5, 7.5]	7	[3,7]	>0.05	5	[5, 12]	5.5	[3, 7]	>0.05
Endometrial thickness on the Day of hCG (mm)	9.9	[9.3, 11.5]	11.5	[11, 13]	>0.05	12	[10, 12]	9.6	[8.5, 11]	>0.05

No of oocytes	5	[4, 8]	6	[4, 7]	>0.05	5	[3.5, 7.5]	5	[4, 7]	>0.05
No of MII oocytes	4.5	[4, 5]	4	[3, 6]	>0.05	5	[2, 7]	4	[3, 5]	>0.05
4 oocytes (N, %)	2	29%	7	29%	>0.05	4	50%	11	34%	>0.05
4/9 oocytes (N, %)	5	71%	17	71%	>0.05	4	50%	21	66%	>0.05
ET 1 BC (N, %)	0	0%	1	4%	>0.05	2	25%	11	34%	>0.05
ET 2BC (N, %)	3	43%	7	29%	>0.05	3	38%	5	16%	>0.05
ET -BC, 1BC or 2 BC (N, %)	3	43%	8	33%	>0.05	5	63%	16	50%	>0.05
ET 1 oocyte 3rd day (N, %)	0	0%	6	25%	>0.05	0	0%	3	9%	>0.05
ET 2oocytes 3rd day (N, %)	3	43%	8	33%	>0.05	3	38%	7	22%	>0.05
KRIO (N, %)	2	29%	2	8%	>0.05	2	25%	2	6%	>0.05
KRIO 1BC or 2BC (N)	0	[0, 2]	0	[0, 0]	>0.05	0	[0, 1]	0	[0, 0]	>0.05
No. of fresh transferred embryos	2	[2, 2]	2	[1, 2]	>0.05	2	[1.5, 2]	1	[1, 2]	**<0.05**
No. of fresh transferred BC	0	[0, 2]	0	[0, 2]	>0.05	1	[0, 2]	0.5	[0, 1]	>0.05

No. of all BC (ET+FET)	0	[0, 4]	0	[0, 2]	>0.05	1.5	[0, 2.5]	1	[0, 1]	>0.05
Total No of transferred embryos 3rd day	0	[0, 2]	1	[0, 2]	>0.05	0	[0, 2]	0	[0, 1]	>0.05
No. of ET embryos 3rd day	3	43%	14	58%	>0.05	3	38%	10	31%	>0.05

Categorical variables are represented as absolute frequency (N) and relative frequency (p, %) and were compared using the chi–square test. Continuous variables are described as medians with [first quartile (Q1), third quartile (Q3)], and were compared using the Mann–Whitney U test. Statistically significant differences are indicated in bold. BC, blastocyst; 1 BC, one blastocyst; 2BC, two blastocysts; ET, embryo transfer; KRIO, frozen embryo; FET, frozen embryo transfer; GnRH-ant, gonadotropin-releasing hormone antagonist; hCG, Human Chorionic Gonadotropin; MII, metaphase II.
